# A G4P[13] Porcine Rotavirus a Strain with Genomic Features Suggestive of Reassortment: Isolation, Genomic Characterization, and Pathogenicity in Piglets

**DOI:** 10.3390/vetsci13090907

**Published:** 2026-09-04

**Authors:** Xianyu Zhang, Rui Geng, Shengjin Liu, Liguo Gao, Qunhui Li, Yongchang Cao, Yu Wu, Hanqin Shen

**Affiliations:** 1 School of Food & Pharmaceutical Engineering, Zhaoqing University, Zhaoqing 526061, China; 2010020066@zqu.edu.cn; 2 State Key Laboratory of Biocontrol, School of Life Sciences, Sun Yat-sen University, Guangzhou 510275, Chinacaoych@mail.sysu.edu.cn (Y.C.); 3 College of Animal Science, South China Agricultural University, Guangzhou 510642, China; 4 Guangdong Provincial Enterprise Key Laboratory of Healthy Animal Husbandry and Environment Control, Wen’s Foodstuff Group Co., Ltd., Yunfu 527400, China

**Keywords:** porcine rotavirus, RVA, G4P[13], pathogenicity

## Abstract

Porcine rotavirus A (PoRVA) is an important cause of diarrhea in neonatal piglets, resulting in significant economic losses in the swine industry. In this study, a PoRVA strain, designated QY, was isolated from diarrheic piglets and systematically characterized. The virus was propagated in MA104 cells and identified by immunofluorescence assay and transmission electron microscopy, showing typical cytopathic effects and spherical particles of approximately 70 nm. Whole-genome sequencing and phylogenetic analysis revealed that QY was a G4P[13] strain with a genotype constellation of G4–P[13]–I1–C1–M1–R1–A8–N1–T1–E1–H1. Several genome segments showed close phylogenetic relationships with human- or bat-associated RVA strains, a pattern consistent with a possible reassortment history of QY. Experimental infection showed that QY caused watery diarrhea, intestinal lesions, and viral shedding in neonatal piglets under the experimental conditions used in this study. High viral loads were detected in small intestinal tissues, accompanied by villous atrophy and epithelial degeneration. These findings provide new insights into the genetic diversity and evolution of PoRVA and further characterize the pathogenicity of the G4P[13] QY strain in neonatal piglets.

## Simple Summary

Diarrhea in newborn piglets is an important disease that causes economic losses in the swine industry. In this study, we identified and characterized a new rotavirus strain from diarrheic piglets in China. The virus showed unique genetic features compared with previously reported pig rotaviruses and was able to cause diarrhea, intestinal damage, and virus shedding in young pigs. These findings improve our understanding of the diversity and evolution of rotaviruses in pigs and highlight the importance of continuous monitoring of emerging viral strains to support disease prevention and control.

## 1. Introduction

Rotavirus (RV) is a major etiological agent of viral gastroenteritis in human infants as well as in young animals, including piglets, calves, and lambs [1,2,3]. Rotavirus belongs to the genus *Rotavirus* within the family *Sedoreoviridae* and is a non-enveloped virus with a segmented double-stranded RNA genome [2,4]. The viral particle exhibits an icosahedral structure with a diameter of approximately 70–80 nm and consists of three concentric protein layers encapsulating 11 genome segments. The intermediate capsid protein VP6 is highly conserved and forms the basis for classification into distinct antigenic groups (A–J) [2]. Among these, RVA has the widest host range and is the predominant rotavirus species associated with enteric diseases in both humans and animals. In pigs, PoRVA infection is commonly associated with neonatal diarrhea, leading to intestinal dysfunction, growth retardation, and economic losses in swine production. Therefore, continuous surveillance and characterization of emerging PoRVA strains are essential for understanding viral evolution and improving disease control strategies.

Based on the complete genome constellation, RV strains are classified according to the Gx–P[x]–Ix–Rx–Cx–Mx–Ax–Nx–Tx–Ex–Hx nomenclature, with each segment encoding a specific viral protein, including six structural proteins (VP1–VP4, VP6, and VP7) and five or six non-structural proteins (NSP1–NSP5/6) [4,5]. Among these, VP7 and VP4, located on the outer capsid, play critical roles in viral infectivity and host immune responses [6].

Porcine rotavirus was first isolated from diarrheic piglets in 1976 [4,7]. Infection with PoRVA typically results in diarrhea, anorexia, dehydration, and growth retardation [3,8,9]. In recent years, PoRVA has been prevalent in swine herds in China and has been frequently detected in co-infection with porcine epidemic diarrhea virus (PEDV), posing a substantial threat to the swine industry [10,11,12,13]. Epidemiological studies have shown that G9, G5, and G4 genotypes, together with P[23], P[13], and P[6], are currently predominant in Chinese swine populations [10,11,12,13].

In the present study, a G4P[13] PoRVA strain, designated QY, was isolated from diarrheic piglets on a commercial pig farm in Guangdong Province, China. The biological characteristics of this isolate were investigated, and its pathogenicity was evaluated in 3-day-old piglets. These findings provide valuable insights into the epidemiology and control of PoRVA infections.

## 2. Materials and Methods

### 2.1. Sample Collection and Detection of PoRVA

In March 2025, severe diarrhea occurred in 3–7-day-old piglets on a commercial breeding farm in Guangdong, China. Intestinal tissue samples were collected from eight diarrheic piglets for etiological investigation. All eight samples tested positive for PoRVA, whereas PEDV, TGEV, PDCoV, and SADS-CoV were not detected. A PoRVA-positive intestinal sample was subsequently used for virus isolation, and the successfully isolated QY strain was subjected to whole-genome sequencing and further characterization. Samples were homogenized in PBS and centrifuged at 12,000× *g* for 10 min, and the supernatants were used for viral nucleic acid extraction. Routine screening for enteric viruses, including PEDV, TGEV, PDCoV, PoRVA, and SADS-CoV, was performed by real-time RT-PCR as previously described [10,14]. Total RNA/DNA was extracted using the HiPure Viral DNA/RNA Kit (Magen, Guangzhou, China) according to the manufacturer’s instructions. PoRVA was detected using VP6-specific primers and probe (PoRV-F: GGA CTT ACA TTA CGA ATT GAA TCT G; PoRV-R: TTG CCA AYA AAG TTT CRG AAG C; probe: AGT TTG TGA ATC TGT GCT TGC GGA) [14]. Detection of PoRVA, PEDV, TGEV, PDCoV, and SADS-CoV was further performed using a one-step real-time RT-PCR kit (Q225, Vazyme, China) under standard cycling conditions.

### 2.2. Virus Isolation

MA104 cells (ATCC CRL-2378) were maintained in DMEM supplemented with 5% fetal bovine serum (FBS; Gibco, Grand Island, NY, USA). For PoRVA isolation, the supernatant of the above sample was filtered through a 0.45 μm membrane and inoculated onto confluent MA104 monolayers in six-well plates. After adsorption for 2 h at 37 °C with 5% CO_2_ in serum-free DMEM containing 1% penicillin–streptomycin and 10 μg/mL trypsin, cells were washed with PBS and maintained in the same medium. Cultures were serially passaged until cytopathic effects (CPE) were observed.

### 2.3. Whole-Genome Sequencing and Phylogenetic Analysis

Whole-genome sequencing was conducted as previous description [3]. Viral RNA was extracted from QY-infected MA104 cell culture supernatants using a commercial viral RNA extraction kit (MAGEN, China) according to the manufacturer’s instructions. The complete genome of PoRVA strain QY was determined using the Illumina MiSeq high-throughput sequencing platform (Illumina, San Diego, CA, USA). Ribosomal RNA depletion was performed using the Ribo-Zero Magnetic Gold Kit (Epicentre Biotechnologies, Madison, WI, USA), and RNA libraries were constructed using the NEBNext^®^ Ultra™ Directional RNA Library Prep Kit (New England Biolabs, Ipswich, MA, USA). The prepared libraries were sequenced using the Illumina MiSeq platform with paired-end sequencing chemistry.

The obtained raw sequencing reads were subjected to quality control and adapter trimming using Trimmomatic v0.39 [15]. The filtered reads were aligned against the *Sus scrofa* reference genome (Sscrofa11.1) using Bowtie2 v2.4.1 [16] to remove host-derived sequences. The remaining clean reads were de novo assembled into contigs using MEGAHIT v1.2.9. The assembled contigs were identified by BLASTn and BLASTx searches against the NCBI nucleotide database and UniProt virus reference database. The complete genome segments of QY were annotated based on comparison with representative rotavirus genome sequences, and the genome sequences were deposited in GenBank under accession numbers PZ347295–PZ347305.

The genotypes of all 11 genome segments were assigned using the RVA genotyping resources implemented in the Bacterial and Viral Bioinformatics Resource Center (BV-BRC; https://www.bv-brc.org/), following the established whole-genome RVA classification framework and standardized Gx–P[x]–Ix–Rx–Cx–Mx–Ax–Nx–Tx–Ex–Hx nomenclature [5]. BLAST analysis was subsequently performed using the NCBI BLAST web server to identify closely related reference sequences for comparative and phylogenetic analyses. Phylogenetic trees for each of the 11 genome segments were constructed using the maximum-likelihood (ML) method implemented in MEGA11 with the General Time Reversible model incorporating a gamma distribution and invariant sites (GTR+G+I). The robustness of the tree topologies was evaluated using 1000 bootstrap replicates, with bootstrap values > 70% considered to indicate strong support. Reference sequences were retrieved from GenBank, and clades were defined based on sequence similarity and phylogenetic clustering.

### 2.4. Immunofluorescence Staining of Virus-Infected Cells

Immunofluorescence assay (IFA) was performed to confirm PoRVA infection according to previously described methods with minor modifications [3]. Briefly, MA104 cells cultured on 6-well plates were infected with QY at an MOI of 0.1 or mock-infected. At 24 h post-infection, cells were fixed with 4% paraformaldehyde and permeabilized with 0.25% Triton X-100. After blocking with 5% bovine serum albumin (BSA), cells were incubated with a mouse anti-PoRVA VP6 monoclonal antibody (Zoonogen, Beijing, China), followed by Alexa Fluor^®^ 488-conjugated goat anti-mouse IgG (Abcam, Cambridge, UK). Fluorescence images were acquired using a fluorescence microscope (Olympus, Hachioji, Japan).

### 2.5. Transmission Electron Microscopy (TEM) Observation

Transmission electron microscopy (TEM) analysis was performed according to previously described methods [3]. Briefly, MA104 cells infected with PoRVA strains QY were collected once cytopathic effects (CPE) were observed in approximately 80–90% of the cell monolayer. Cell suspensions underwent three freeze–thaw cycles, followed by centrifugation at 6000× *g* for 20 min at 4 °C to remove cellular debris. The recovered supernatant was subsequently passed through a 0.22 μm filter and treated with polyethylene glycol 8000 (PEG 8000; Solarbio, Beijing, China) at a final concentration of 10% for overnight precipitation at 4 °C. Viral particles were then sedimented by ultracentrifugation at 100,000× *g* for 2 h at 4 °C. The pellet obtained was resuspended in Tris-buffered saline (TBS), stained with 2% phosphotungstic acid (pH 7.0), and visualized under a transmission electron microscope (Leica, Microsystems, Wetzlar, Germany).

### 2.6. Viral Growth Kinetics

Replication kinetics of QY was evaluated in MA104 cells. Confluent monolayers (~90%) were infected at an MOI of 0.01 and incubated at 37 °C with 5% CO_2_. Culture supernatants were collected at 12, 24, 36, 48, and 60 h post-infection from separate wells, clarified by centrifugation, and stored at −80 °C. Three replicate wells were included at each time point, and viral titers were determined separately for each well. Virus titers were determined by endpoint dilution assay in MA104 cells and calculated as TCID_50_/mL using the Reed–Muench method. Data are presented as the mean ± SD of three technical replicates.

### 2.7. Pathogenicity Assay in Piglets

Colostrum-deprived, clinically healthy 3-day-old piglets (*n* = 10) of Duroc × Landrace × Yorkshire crossbred origin were obtained from the same litter on a commercial swine farm for the challenge experiment. Prior to inoculation, fecal samples from all piglets were screened by qRT-PCR to confirm the absence of PoRVA. The piglets were randomly assigned to two groups (*n* = 5 per group): a challenge group and a mock-inoculated control group. Piglets in the two groups were housed separately in disinfected individual isolation units throughout the study period. All piglets were provided with commercial milk replacer at 4-h intervals. At 3 days of age, piglets in the challenge group were orally inoculated with 2 mL of the PoRVA QY strain containing a total dose of 2 × 10^6^ TCID_50_, whereas piglets in the control group received an equivalent volume of virus-free cell culture supernatant via the same route. General clinical status, including physical appearance, behavior, feed intake, and fecal characteristics, was monitored daily throughout the 7-day observation period. The severity of diarrhea was evaluated using a standardized scoring system ranging from 0 to 4: score 0, normal feces; score 1, pasty feces; score 2, semi-liquid feces; score 3, mucoid diarrhea; and score 4, profuse watery diarrhea. Rectal swabs were collected from each surviving piglet daily for viral RNA extraction. Viral shedding was quantified by qPCR as described above, and genome copy numbers were calculated according to a standard curve established using a recombinant plasmid harboring the PoRVA VP6 gene [3]. At 7 dpi, all surviving piglets were euthanized, and intestinal tissues, including the duodenum, jejunum, and ileum, were collected immediately for viral load determination, histopathological examination, and immunohistochemical analysis. Because the piglets were neonatal (3 days old at inoculation) and the experimental period was limited to 7 days, body weight change was not included as an outcome measure in this study.

### 2.8. Histopathology and Immunohistochemistry

Histopathological examination was performed following previously described procedures [14]. At necropsy, tissue samples from the duodenum, jejunum, and ileum were collected from piglets and fixed in 4% paraformaldehyde at room temperature for 48 h. Following fixation, the samples were processed using routine histological procedures, including dehydration through a graded ethanol series, clarification in xylene, and paraffin embedding. Paraffin-embedded tissues were sectioned at an appropriate thickness using a microtome (Leica). The sections were subsequently deparaffinized in xylene and rehydrated through descending concentrations of ethanol. For histopathological evaluation, tissue sections were stained with hematoxylin and eosin (H&E; BaSo, Zhuhai, China) according to standard protocols and examined under a light microscope (Olympus, Hachioji, Japan). For immunohistochemical (IHC) analysis, the sections were subjected to antigen retrieval when required and then incubated with a mouse monoclonal antibody against the PoRVA VP6 protein (Zoonogen, Beijing, China). After primary antibody incubation, sections were treated with a corresponding secondary antibody and visualized using a commercial detection system following the manufacturer’s instructions. Finally, the sections were counterstained with hematoxylin and examined microscopically. Comparative analysis between infected and control groups was performed to evaluate tissue lesions and the distribution of viral antigens.

### 2.9. Statistical Analysis

Data were processed and plotted using GraphPad Prism version 10.0 (GraphPad Software, San Diego, CA, USA). Data were processed and plotted using GraphPad Prism version 10.0 (GraphPad Software, San Diego, CA, USA). Because the animal challenge experiment was designed as an exploratory pathogenicity study rather than a comparative efficacy study, clinical scores, viral shedding, intestinal viral loads, and survival outcomes were summarized descriptively. Data are presented as the mean ± standard deviation (SD), where applicable. Longitudinal clinical scores and viral shedding were interpreted descriptively, and missing observations resulting from mortality were not imputed. No formal between-group hypothesis testing was performed for these outcomes. Statistical significance was therefore not inferred unless specifically indicated.

## 3. Results

### 3.1. Virus Isolation, Identification, and Growth Curves of QY

**Figure 1:**
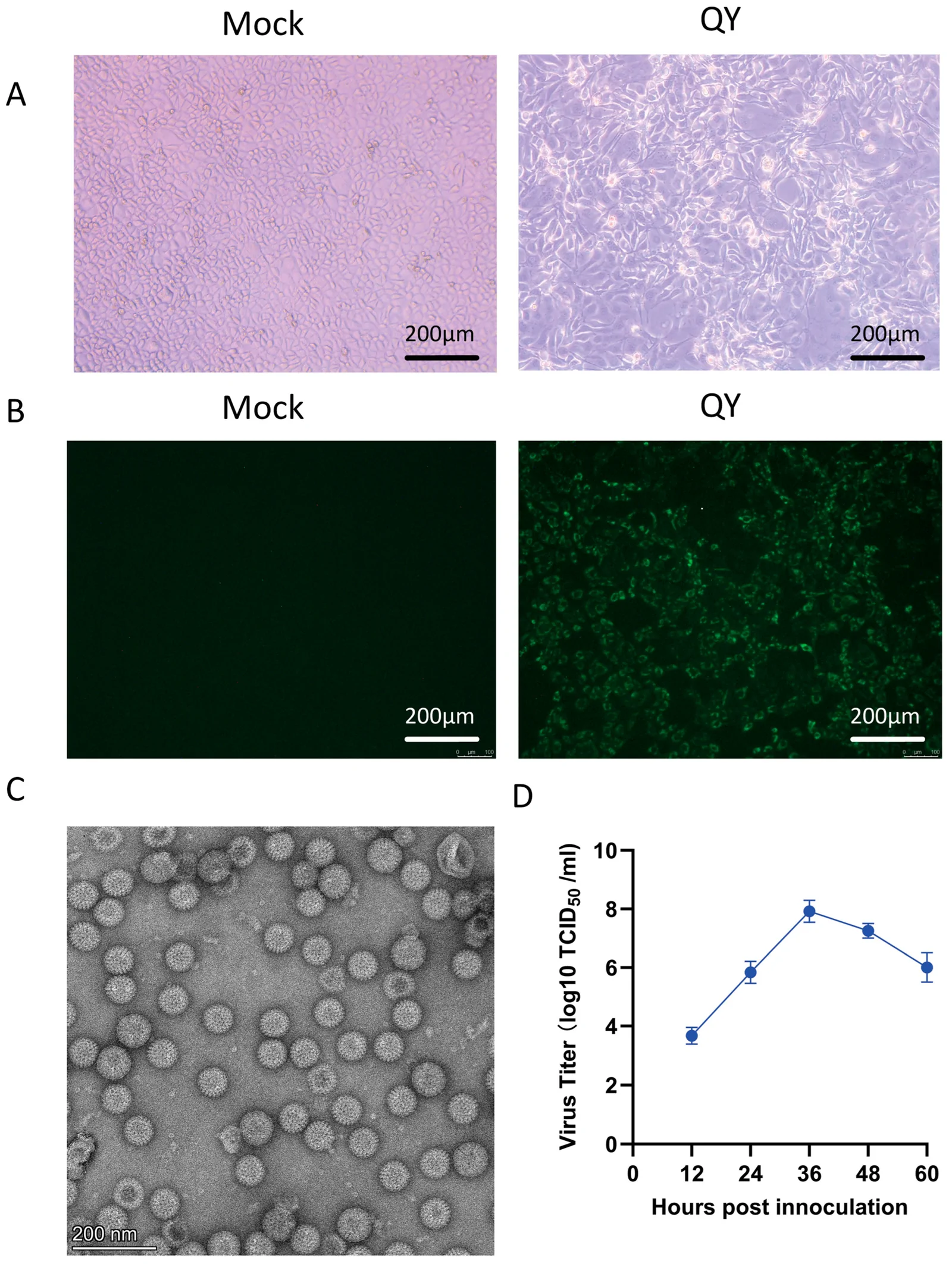
In vitro characterization of porcine group A rotavirus (PoRVA) strain QY. (**A**) Cytopathic effects (CPE) observed in MA104 cells (scale bar = 200 μm). (**B**) Immunofluorescence assay of QY-infected MA104 cells using anti-VP6 antibodies. (**C**) Transmission electron microscopy showing viral particles of QY (scale bar = 200 nm). (**D**) Multi-step growth curve of QY in MA104 cells determined by TCID_50_ assay. The viral growth curve was determined using three technical replicates at each time point. Data are presented as the mean ± SD (*n* = 3), and error bars represent the SD.

All eight intestinal tissue samples collected from diarrheic piglets tested positive for PoRVA and negative for PEDV, TGEV, PDCoV, and SADS-CoV, indicating that no co-infection with these tested enteric viruses was detected. The PoRVA-positive intestinal tissue supernatant was inoculated onto confluent MA104 cell monolayers. After three consecutive blind passages, a viral isolate designated QY was obtained. At 24 h post-infection (hpi) with the third passage of QY, typical cytopathic effects (CPE) were observed in MA104 cells, including cell fusion, fragmentation, and detachment (Figure 1A). The isolate was further identified by immunofluorescence assay (IFA). At 24 hpi, most MA104 cells infected with QY exhibited strong green fluorescence signals (Figure 1B), indicating the presence of rotavirus antigen. Subsequently, the virus was concentrated by ultracentrifugation and examined by negative-staining transmission electron microscopy. As shown in Figure 1C, the QY viral particles displayed a typical spherical morphology with a diameter of approximately 70 nm. The growth kinetics of QY (passage 8, P8) in MA104 cells were then determined by virus titration. As shown in Figure 1D, the virus replicated exponentially before 36 hpi, reaching a peak titer of 8.0 log10 TCID50/mL at 36 hpi, after which the viral titer gradually declined.

### 3.2. Genomic and Phylogenetic Analysis of QY

**Figure 2:**
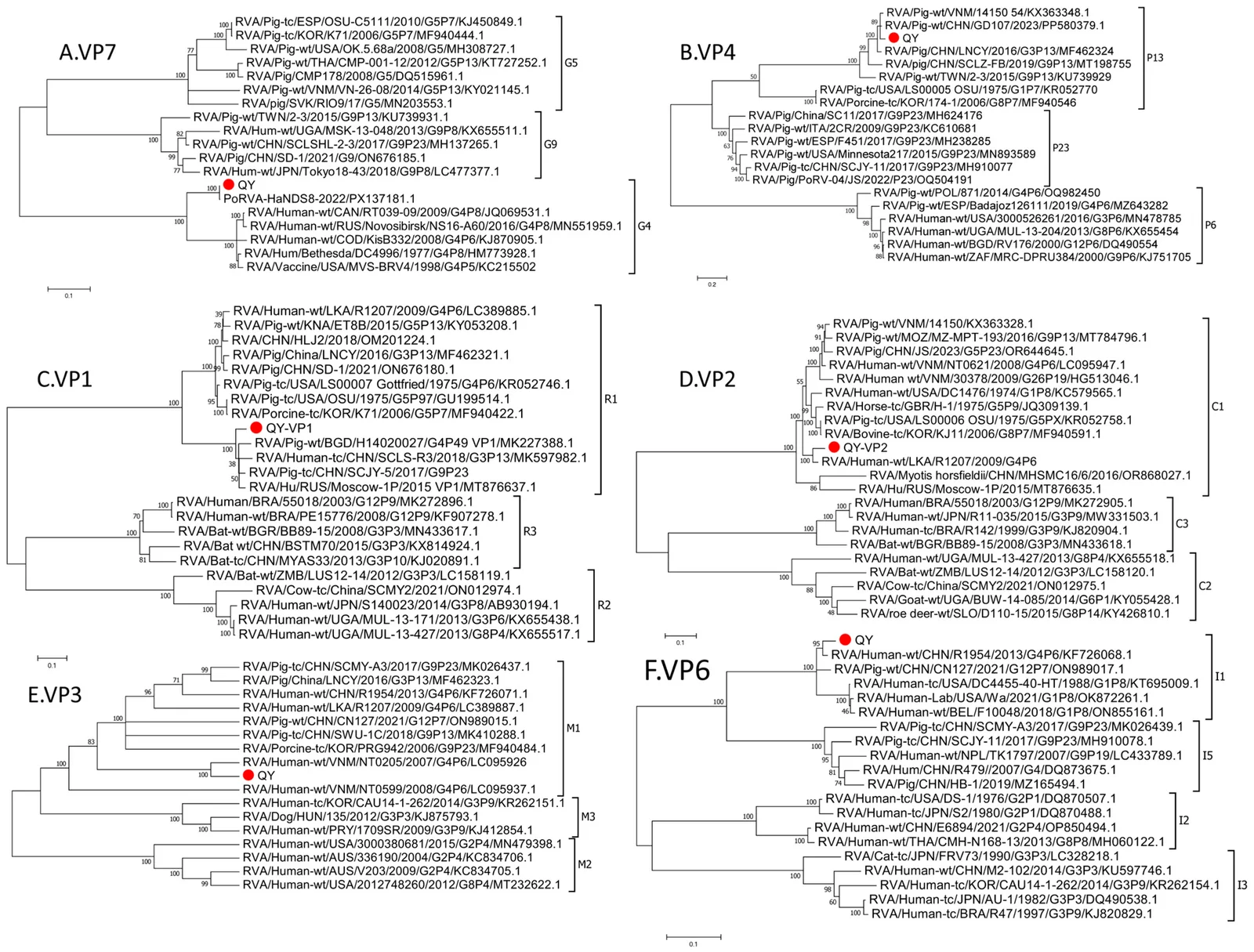
Phylogenetic analysis of structural protein-encoding genes of the QY strain. (**A**) VP7; (**B**) VP4; (**C**) VP1; (**D**) VP2; (**E**) VP3; (**F**) VP6. Phylogenetic trees were constructed based on the open reading frames (ORFs) of the target genes (VP1–VP4 and VP6) identified in this study and reference sequences retrieved from GenBank. Phylogenetic trees were constructed using the maximum-likelihood method with the best-fitting nucleotide substitution model for each genome segment. The reliability of tree topology was assessed using 1000 bootstrap replicates. The QY strain identified in this study is indicated by a red circle. The numbers at the branch nodes indicate bootstrap values, and the red circles indicate the QY strain identified in this study.

**Figure 3:**
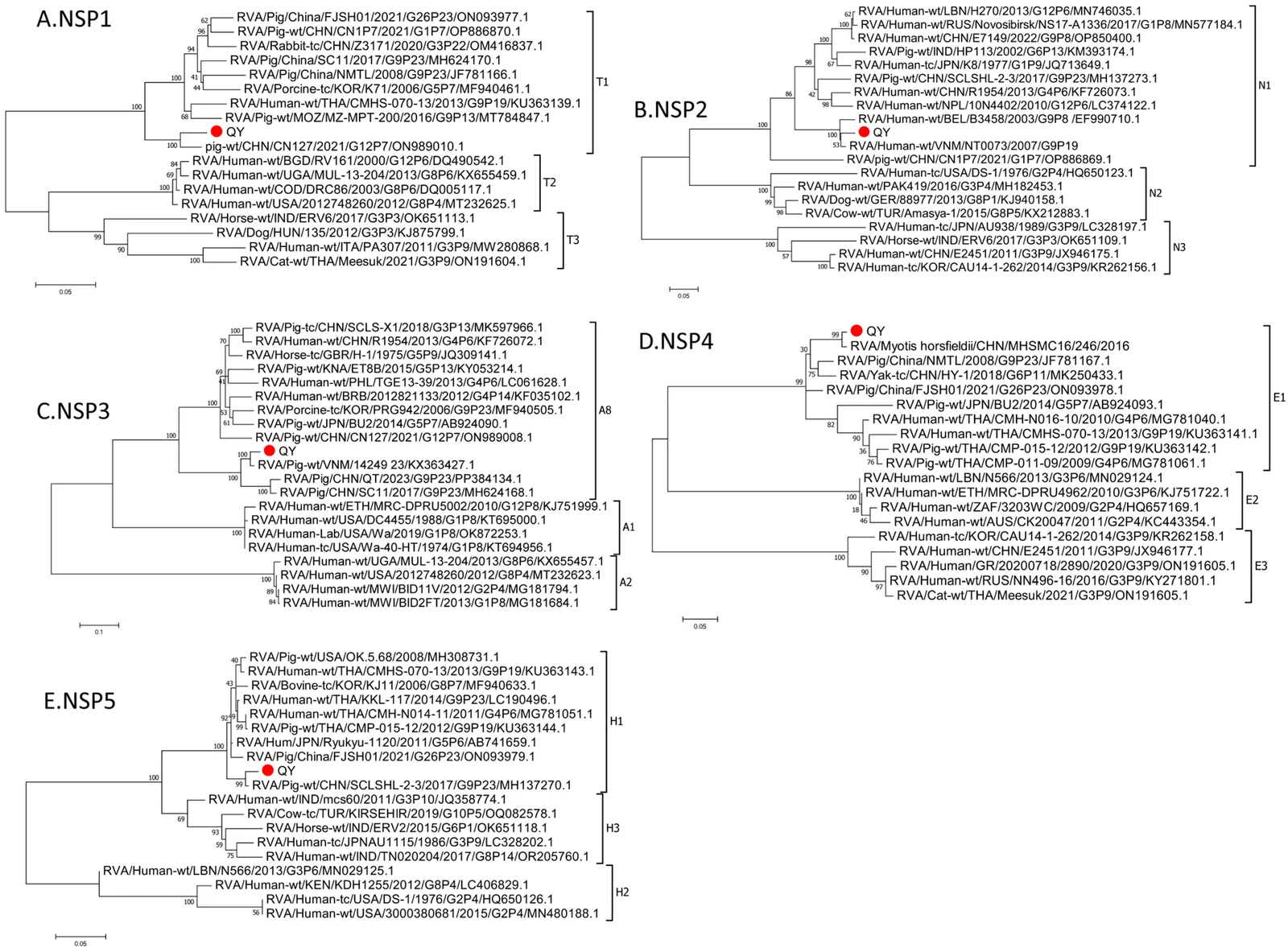
Phylogenetic analysis of non-structural protein-encoding genes of the QY strain. (**A**) NSP1; (**B**) NSP2; (**C**) NSP3; (**D**) NSP4; (**E**) NSP5. Phylogenetic trees were constructed based on the open reading frames (ORFs) of the target genes P5(NSP1-NS) identified in this study and reference sequences retrieved from GenBank. The phylogenetic tree was constructed using the maximum-likelihood method with the GTR+G+I model in MEGA11. Bootstrap values were calculated from 1000 replicates. The numbers at the branch nodes indicate bootstrap values, and the red circles indicate the QY strain identified in this study.

The complete genome sequence of the QY strain was obtained by high-throughput sequencing and deposited in the GenBank database under accession numbers PZ347295–PZ347305. The genotypes of all 11 genome segments were assigned according to the established whole-genome RVA classification framework, and phylogenetic trees were constructed using the maximum-likelihood method. Based on the established whole-genome RVA classification framework, QY was identified as a G4P[13] strain with a whole-genome genotype constellation of G4–P[13]–I1–C1–M1–R1–A8–N1–T1–E1–H1 (Table 1; Figure 2 and Figure 3). At the whole-genome level, the 11 segments of QY showed heterogeneous host-associated genetic relationships. Six segments (VP7, VP4, VP1, NSP1, NSP3, and NSP5) showed the highest nucleotide identities with porcine RVA strains, four segments (VP6, VP2, VP3, and NSP2) were most closely related to human-associated RVA strains, whereas NSP4 showed the highest nucleotide identity with a bat-associated RVA strain (Table 1; Figure 2 and Figure 3). This genome-wide pattern highlights the heterogeneous evolutionary relationships among the genome segments of QY. Further analysis showed that the VP7 gene clustered within the G4 genotype and was closely related to the porcine strain HaNDS8 (Figure 2A), exhibiting 100% sequence identity, while remaining genetically distant from other reference strains. The VP4 gene was classified as the P[13] genotype and exhibited phylogenetic relatedness to multiple P[13] strains circulating in China (Figure 2B), which may reflect a complex evolutionary history. The VP6 gene belonged to the I1 genotype and was closely related to the human-associated strain R1954 (Figure 2F), sharing 97.04% nucleotide identity (Table 1). Notably, the NSP4 gene exhibited the highest sequence identity (98.48%) with strain MHSMC16 (Table 1; Figure 3D), an RVA strain previously detected in *Myotis horsfieldii*. Collectively, these phylogenetic findings reveal heterogeneous evolutionary relationships among the genome segments of QY and are consistent with a possible reassortment history. However, the timing, direction, and host species involved in any putative reassortment event cannot be determined from the present phylogenetic evidence. Nevertheless, alternative explanations, including incomplete sampling of circulating RVA diversity, shared evolutionary ancestry, and limited availability of closely related reference sequences, cannot be excluded.

### 3.3. Pathogenicity in 3-Day-Old Piglets

**Figure 4:**
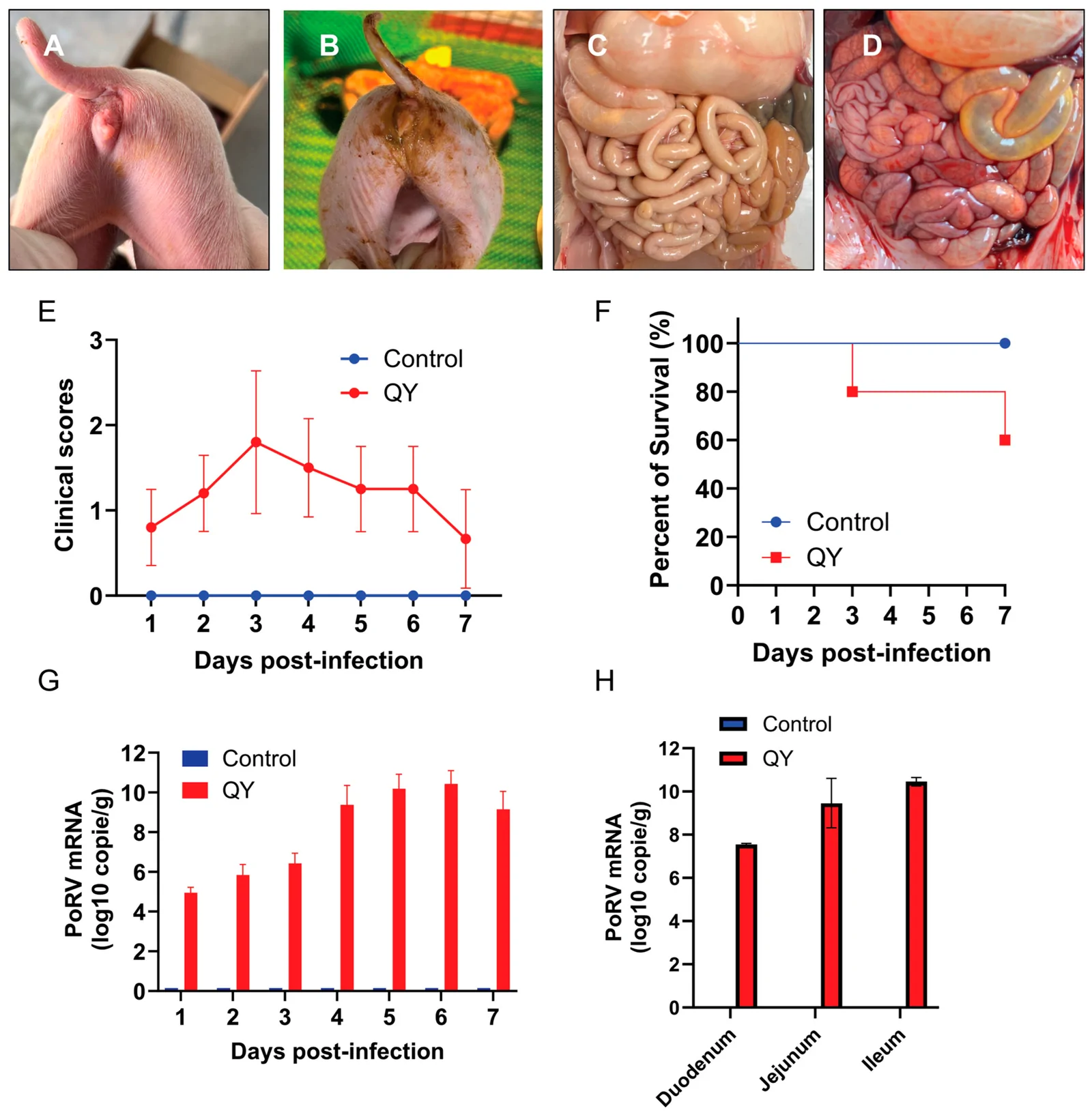
Pathological lesions and pathogenicity of piglets infected with the PoRVA strain QY. (**A**) Representative appearance of a mock-inoculated control piglet without diarrhea; (**B**) Representative appearance of a QY-infected piglet showing watery diarrhea; (**C**) Representative gross intestinal morphology of a mock-inoculated control piglet; (**D**) Representative gross intestinal lesions of a QY-infected piglet; (**E**) Clinical scores of piglets following QY challenge (n = 5 per group at the start of the experiment); (**F**) Survival curves of piglets after challenge (n = 5 per group); (**G**) Viral shedding in rectal swabs (n = 5 per group at the start of the experiment); (**H**) Viral loads in the duodenum, jejunum, and ileum of surviving QY-infected piglets collected at 7 dpi (n = 3). Data are presented as the mean ± SD where applicable.

To evaluate the pathogenicity of the QY strain, five 3-day-old piglets were orally inoculated with 2 mL of QY (passage 8, P8) at a dose of 1 × 10^6^ TCID_50_/mL, while another five piglets received an equal volume of virus-free cell culture supernatant as mock-inoculated controls. Piglets in the challenged group developed diarrhea at 2 days post-infection (dpi), which peaked at 3 dpi and was characterized by typical watery diarrhea (Figure 4B). The severity of diarrhea gradually decreased thereafter (Figure 4E). Two of the five challenged piglets died during the experiment, one at 3 dpi and the other at 7 dpi, corresponding to an observed survival rate of 60% (Figure 4F). Both piglets exhibited severe diarrhea before death. However, necropsy was not performed on these animals; therefore, the definitive cause of death could not be determined. At 7 dpi, necropsy revealed typical gross lesions in the challenged group, including thinning and translucency of the intestinal wall, as well as accumulation of yellow fluid within the intestinal lumen (Figure 4D). Viral shedding in rectal swabs was quantified by real-time RT-PCR. All challenged piglets tested positive as early as 24 h post-infection (hpi) and remained positive until the end of the experiment, with peak viral shedding observed at 4 dpi (Figure 4G). At necropsy (7 dpi), small intestinal tissues were collected for viral load determination. High levels of viral RNA were detected in the duodenum, jejunum, and ileum (Figure 4H).

### 3.4. Intestinal Histopathology in Piglets Infected with QY

**Figure 5:**
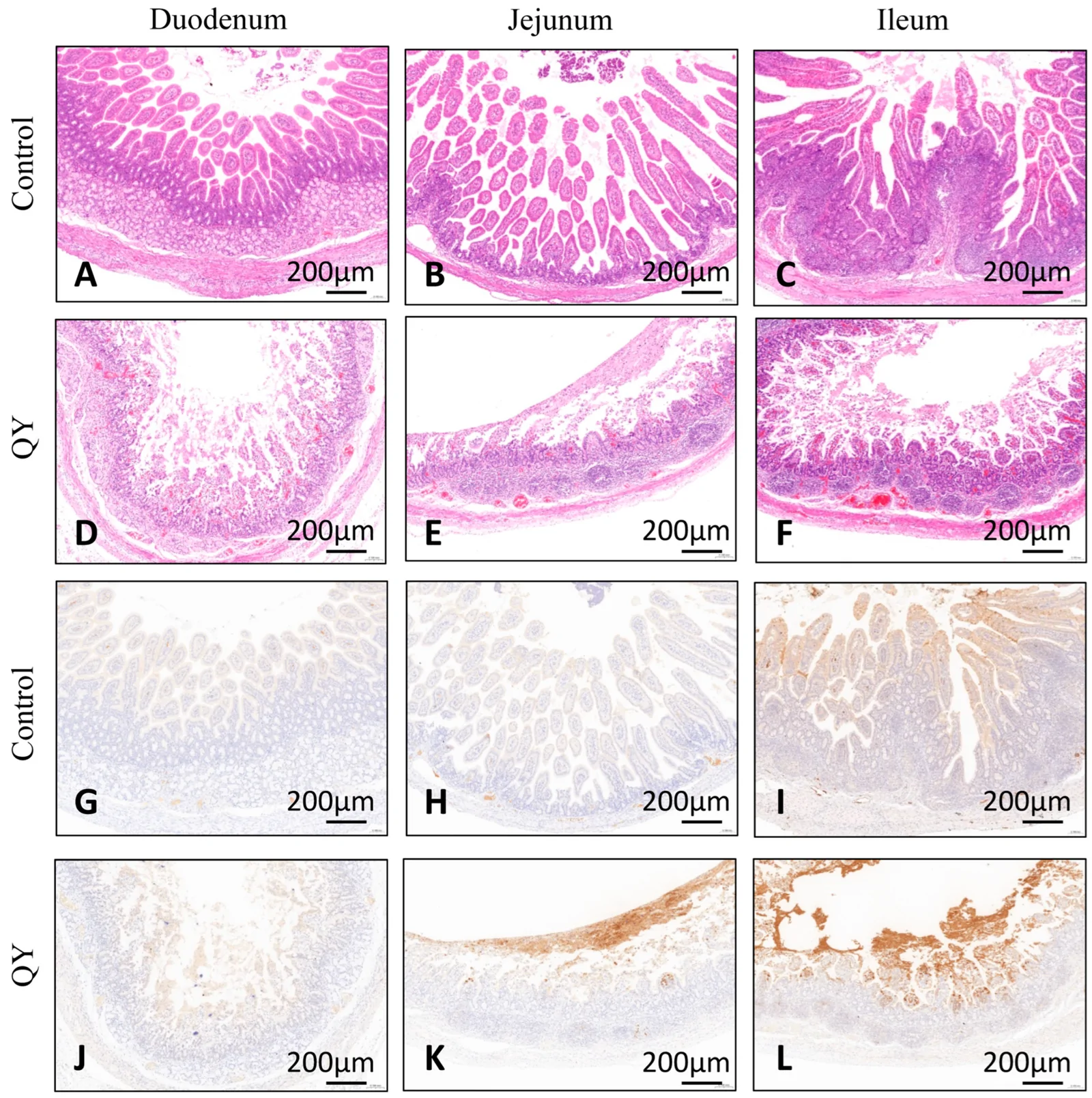
Histopathological and immunohistochemical (IHC) analysis of the small intestine in piglets infected with the PoRVA strain QY. (**A**–**C**) Representative hematoxylin and eosin-stained sections of the duodenum (**A**), jejunum (**B**), and ileum (**C**) from mock-inoculated control piglets; (**D**–**F**) corresponding intestinal sections from QY-infected piglets; (**G**–**I**) IHC staining of the duodenum (**G**), jejunum (**H**), and ileum (**I**) from mock-inoculated control piglets showing no specific signal; (**J**–**L**) IHC staining of the duodenum (**J**), jejunum (**K**), and ileum (**L**) from QY-infected piglets showing VP6-positive signals in intestinal epithelial cells and some mononuclear cells. Representative images were obtained from three piglets per group (n = 3). All tissue sections were examined at ×100 magnification. Scale bar = 200 μm.

Histopathological examination was conducted at 7 days post-infection (dpi). Intestinal tissues from the control group exhibited normal histological architecture, and no PoRVA antigen was detected by immunohistochemistry (IHC). In contrast, piglets in the challenged group showed varying degrees of intestinal mucosal atrophy, villous epithelial degeneration and necrosis, villous destruction, vascular congestion in the lamina propria, and inflammatory cell infiltration. Specifically, the duodenum exhibited moderate mucosal atrophy with mild epithelial degeneration and necrosis, accompanied by slight vascular congestion and lymphocytic infiltration (Figure 5D). IHC-positive signals were mainly observed in exfoliated villous epithelial cells (Figure 5G). The jejunum showed more pronounced lesions, including moderate mucosal atrophy, evident epithelial cell degeneration and necrosis with sloughing into the intestinal lumen, and mild infiltration of inflammatory cells (lymphocytes, plasma cells, and macrophages) (Figure 5E). IHC-positive signals were detected in villous epithelial cells and in some mononuclear cells within the lamina propria and Peyer’s patches (Figure 5K). In the ileum, similar but slightly milder lesions were observed, including mucosal atrophy, epithelial degeneration and necrosis, vascular congestion, and mild inflammatory infiltration (Figure 5F). Positive IHC signals were observed in villous epithelial cells and some mononuclear cells in the lamina propria (Figure 5L). Overall, these findings indicate that the jejunum and ileum are the primary target sites of PoRVA infection in the small intestine.

## 4. Discussion

Porcine rotavirus A remains a major enteric pathogen causing diarrhea in neonatal piglets and continues to pose a significant threat to the swine industry. PoRVA is widely distributed in swine populations in China, with G9, G5, and G4 being the predominant circulating genotypes [10,12]. While extensive studies have been conducted on G9 and G5 strains, including virus isolation, pathogenicity evaluation, and vaccine development [9,17,18], systematic investigations on the pathogenicity of G4 genotype strains remain limited [19,20]. In the present study, a PoRV G4P[13] strain, QY, was successfully isolated and characterized, and its biological properties and pathogenicity were systematically evaluated. The results showed that QY induced typical cytopathic effects in MA104 cells and reached high viral titers within a short period, indicating strong in vitro replication capacity. These findings provide a basis for further investigation of the immunogenicity, antigenic characteristics, safety, and protective efficacy of QY, which will be necessary before its potential application in vaccine development can be evaluated [10,18].

Genomic analysis demonstrated that QY belongs to the G4P[13] genotype currently circulating in swine populations in China [12]. When the complete 11-segment genome constellation was considered, QY showed heterogeneous genetic relationships rather than a uniformly porcine-like genomic background. Six genome segments were most closely related to porcine RVA strains, four segments showed the closest relationships with human-associated RVA strains, and NSP4 was most closely related to a bat-associated RVA strain. Notably, the VP6 gene was classified as the I1 genotype and showed high similarity to the human RVA strain R1954, whereas the I5 genotype is predominant among porcine RVA strains circulating in China [18]. VP6 is a highly conserved inner capsid protein involved in viral genome replication, transcription, and particle assembly. The detection of an I1 VP6 genotype in a porcine RVA strain suggests a distinct evolutionary history of this segment and is consistent with a possible reassortment history. Although I1 VP6 strains have occasionally been detected in animal populations, the predominance of I5 among Chinese porcine RVA strains indicates that QY possesses a distinct genomic feature compared with most previously reported porcine RVA strains. Similarly, the close relationships of the VP2, VP3, and NSP2 segments with human-associated RVA strains further indicate heterogeneous evolutionary relationships among the genome segments of QY, which are consistent with a possible reassortment history. Because RVA possesses an 11-segment double-stranded RNA genome, reassortment can readily occur during co-infection and represents an important mechanism driving viral genetic diversity and adaptation [21]. In addition, the NSP4 gene of QY was most closely related to a *Myotis horsfieldii*-derived rotavirus. NSP4 functions as a viral enterotoxin and contributes to rotavirus-induced diarrhea through regulation of intracellular calcium signaling. However, the phylogenetic relationship between QY NSP4 and the bat-associated RVA strain does not establish a bat origin of this segment or provide evidence for direct bat-to-pig transmission. This relationship may instead reflect shared evolutionary ancestry, historical genetic exchange among RVA populations, or incomplete sampling of circulating RVA diversity. Collectively, this incongruent genome-wide pattern is consistent with a possible reassortment history. However, the present sequence and phylogenetic evidence does not allow identification of the exact parental strains or determination of the direction, timing, or host species involved in any putative reassortment event. Incomplete sampling of circulating RVA diversity and the limited availability of closely related complete genomes should also be considered when interpreting the evolutionary history of QY.

In addition to their evolutionary significance, VP4 and VP7 are the two major outer capsid proteins of RVA and are important targets of neutralizing antibodies. VP4 is involved in virus attachment and entry, whereas both VP4 and VP7 contain neutralizing epitopes that contribute to the induction of protective antibody responses [22]. Genetic and antigenic variation in these outer capsid proteins may influence susceptibility to neutralizing antibodies and cross-neutralization among RVA strains [23]. However, sequence variation or phylogenetic divergence alone is insufficient to demonstrate immune escape or reduced vaccine protection. Therefore, antigenic mapping, cross-neutralization assays, and vaccine challenge studies will be required to determine whether the VP4 and VP7 characteristics of QY affect its antigenic properties or recognition by antibodies induced by existing vaccine strains.

Pathogenicity analysis demonstrated that QY is highly virulent in neonatal piglets, causing typical watery diarrhea, intestinal lesions, and sustained viral shedding. Although two of the five challenged piglets died, the limited sample size precludes a definitive assessment of the mortality associated with QY. Nevertheless, the observed diarrhea, intestinal lesions, viral shedding, and intestinal viral loads demonstrate its pathogenic potential in neonatal piglets. Histopathological examination revealed characteristic lesions associated with rotavirus infection, including villous atrophy, epithelial degeneration and necrosis, and inflammatory cell infiltration. Notably, lesions in the jejunum were more severe, which is consistent with previous studies indicating that rotavirus preferentially infects mature enterocytes in the small intestine [3,9]. Immunohistochemical analysis showed that viral antigens were predominantly detected in intestinal epithelial cells and were also observed in some mononuclear cells within the lamina propria and Peyer’s patches. However, the presence of VP6-positive signals alone does not demonstrate productive viral infection or replication in these mononuclear cells. Their cellular identity and the biological significance of the detected viral antigen require further investigation using cell-specific markers and co-localization assays. In addition, sustained viral shedding was observed in experimentally infected piglets; however, whether QY can be efficiently transmitted under field conditions remains to be determined.

Despite the comprehensive characterization of the genetic features and pathogenicity of QY in this study, several limitations remain. For instance, the immunogenicity, antigenic characteristics, safety, and protective efficacy of this strain were not evaluated in the present study, and its potential for interspecies transmission was not experimentally verified. Although all eight intestinal samples collected during the outbreak were positive for PoRVA and negative for PEDV, TGEV, PDCoV, and SADS-CoV, these samples originated from a single commercial farm, and only the isolated QY strain was subjected to whole-genome sequencing. Therefore, the prevalence and geographic distribution of G4P[13] PoRVA strains cannot be inferred from the present study. Broader epidemiological surveillance involving multiple farms and geographic regions is required to determine the prevalence and distribution of this genotype. One limitation of the present study is that clinical scoring and histopathological evaluation were not performed in a blinded manner. However, the pathological findings were supported by immunohistochemical staining and quantitative viral load analyses, both of which yielded consistent results. Future studies will incorporate blinded assessment procedures to further strengthen the rigor of animal experiments.

One limitation of the present study is that clinical scoring and histopathological evaluation were not performed in a blinded manner. However, the pathological findings were supported by immunohistochemical staining and quantitative viral load analyses, which yielded consistent results. Future studies will incorporate predefined quantitative lesion-scoring criteria and blinded independent evaluation to further strengthen the rigor of pathological assessment. In addition, because only five piglets were included in each group, the observed mortality and pathogenicity should be interpreted cautiously, and challenge studies involving larger numbers of animals are required to more accurately characterize these outcomes. The use of piglets from a single litter reduced variation in maternal and early-life background but may have introduced a potential litter effect; therefore, future studies involving piglets from multiple litters are warranted to further validate these findings. Because the animal experiment was terminated at 7 days post-infection, the duration of viral shedding beyond this time point could not be determined, and longer observation periods will be required to define the complete viral shedding profile of QY. Furthermore, the evolutionary origin of QY was inferred primarily from phylogenetic relationships and sequence similarity of individual genome segments. Although these findings suggest a complex evolutionary history and are consistent with a possible reassortment history, additional comparative genomic analyses, expanded genomic surveillance, and broader epidemiological investigations will be required to further clarify the evolutionary origin of this strain.

## 5. Conclusions

In conclusion, QY is a G4P[13] PoRVA strain that exhibited clear pathogenicity in neonatal piglets under the experimental conditions used in this study, while its genomic characteristics are consistent with a possible reassortment history. Larger-scale animal studies and expanded molecular surveillance are required to further evaluate its pathogenicity and evolutionary origin.

## Figures and Tables

**Table 1:** Nucleotide sequence identities of QY genome segments compared with closely related reference strains from GenBank.

Gene	Genotype	Closest Strain	Ident %	Accession
VP7	G4	RVA/Pig/CHN/HaNDS8/2022	100.00%	PX137181.1
VP4	P[13]	RVA/Pig-wt/VNM/14150_54/2012	96.52%	KX363348.1
VP6	I1	RVA/Human-wt/CHN/R1954/2013/G4P[6]	97.04%	KF726068.1
VP1	R1	RVA/Pig-tc/CHN/SCJY-5/2017/G9P[23]	95.78%	MH898987.1
VP2	C1	RVA/Human-wt/LKA/R1207/2009/G4P[6]	95.66%	LC389886.1
VP3	M1	RVA/Human-wt/VNM/NT0205/2007/G4P[6]	93.57%	LC095926.1
NSP1	A8	RVA/Pig-wt/VNM/14249_23/2012	96.74%	KX363427.1
NSP2	N1	RVA/Human-wt/VNM/NT0073/2007/G9P[19]	97.06%	LC095906.1
NSP3	T1	RVA/pig-wt/CHN/CN127/2021/G12P[7]	96.36%	ON989010.1
NSP4	E1	RVA/Myotis horsfieldii/CHN/MHSMC16/246/2016	98.48%	OR868018.1
NSP5	H1	RVA/Pig-wt/CHN/SCLSHL-2-3/2017/G9P[23]	98.61%	MH137270.1

## Data Availability

The original contributions presented in this study are included in the article. Further inquiries can be directed to the corresponding author(s).

## Abbreviations

The following abbreviations are used in this manuscript:

RVA	Rotavirus A
PoRVA	Porcine rotavirus A
VP	Viral protein
NSP	Nonstructural protein
CPE	Cytopathic effect
IFA	Immunofluorescence assay
TEM	Transmission electron microscopy
WGS	Whole-genome sequencing
MOI	Multiplicity of infection
TCID50	50% Tissue culture infectious dose
dpi	Days post-infection
hpi	Hours post-infection
qRT-PCR	Quantitative reverse transcription polymerase chain reaction
IHC	Immunohistochemistry
H&E	Hematoxylin and eosin
